# Examination of Csr regulatory circuitry using epistasis analysis with RNA-seq (Epi-seq) confirms that CsrD affects gene expression via CsrA, CsrB and CsrC

**DOI:** 10.1038/s41598-018-23713-8

**Published:** 2018-03-29

**Authors:** Anastasia H. Potts, Yuanyuan Leng, Paul Babitzke, Tony Romeo

**Affiliations:** 1 Department of Microbiology and Cell Science, University of Florida, Institute of Food and Agricultural Sciences, Gainesville, Florida 32611-0700 USA; 2 Department of Biochemistry and Molecular Biology and The Pennsylvania State University, University Park, Pennsylvania, 16802 USA; 3 Center for RNA Molecular Biology, The Pennsylvania State University, University Park, Pennsylvania, 16802 USA; 4 Present Address: RNA Biology Laboratory, National Cancer Institute, National Institutes of Health, Frederick, Maryland, 21702 USA

## Abstract

The Csr global regulatory system coordinates gene expression in response to metabolic status. This system utilizes the RNA binding protein CsrA to regulate gene expression by binding to transcripts of structural and regulatory genes, thus affecting their structure, stability, translation, and/or transcription elongation. CsrA activity is controlled by sRNAs, CsrB and CsrC, which sequester CsrA away from other transcripts. CsrB/C levels are partly determined by their rates of turnover, which requires CsrD to render them susceptible to RNase E cleavage. Previous epistasis analysis suggested that CsrD affects gene expression through the other Csr components, CsrB/C and CsrA. However, those conclusions were based on a limited analysis of reporters. Here, we reassessed the global behavior of the Csr circuitry using epistasis analysis with RNA seq (Epi-seq). Because CsrD effects on mRNA levels were entirely lost in the *csrA* mutant and largely eliminated in a *csrB*/*C* mutant under our experimental conditions, while the majority of CsrA effects persisted in the absence of *csrD*, the original model accounts for the global behavior of the Csr system. Our present results also reflect a more nuanced role of CsrA as terminal regulator of the Csr system than has been recognized.

## Introduction

**Figure 1:**
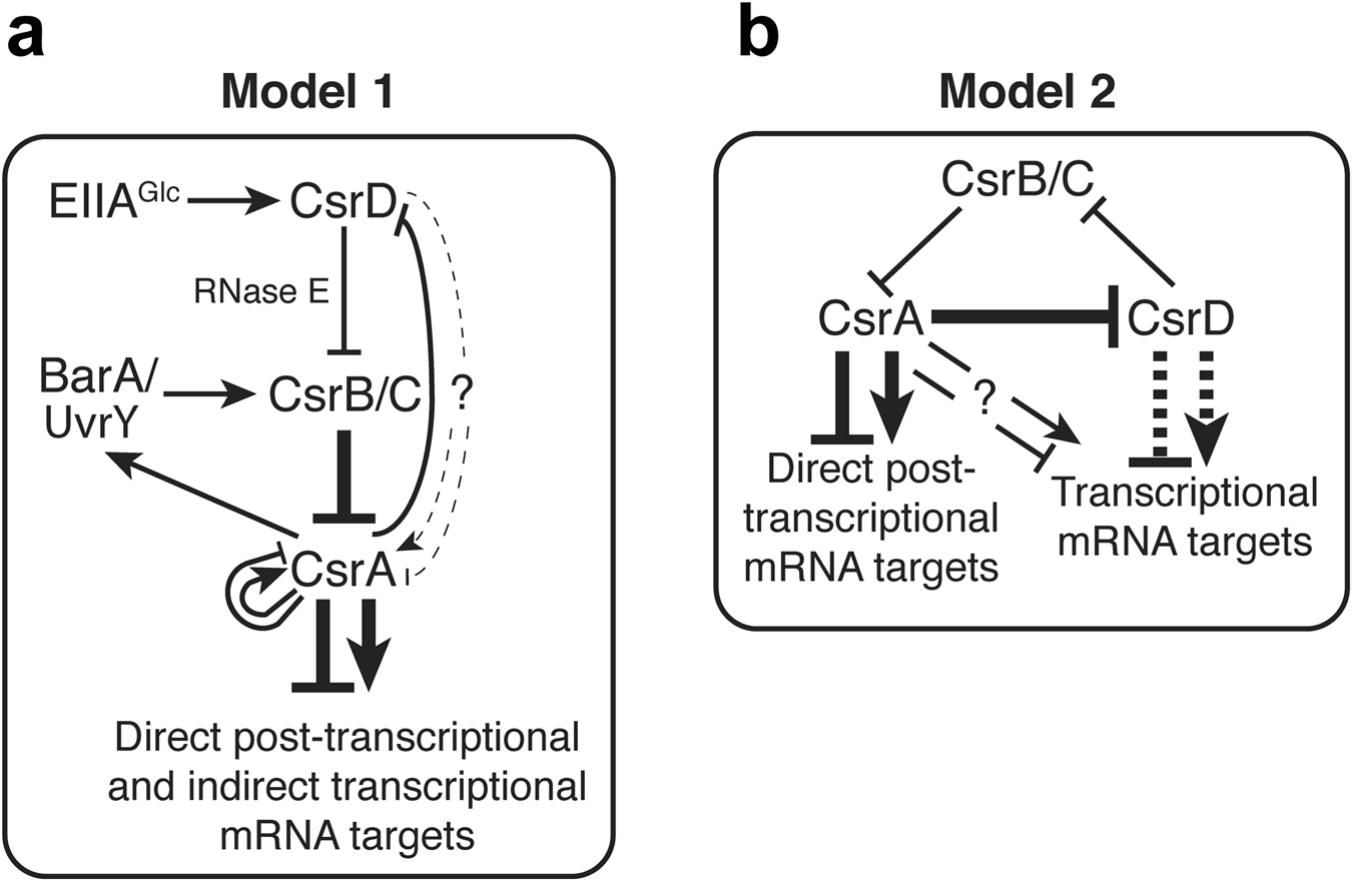
Models proposed for relationships among components of the Csr system. (**a**) Model 1 described in Suzuki *et al*.^26^ and others^1^, wherein CsrD affects gene expression through effects on CsrB/C stability, which affect the ability of CsrA to directly or indirectly control mRNA expression. (**b**) Model 2 proposed by Esquerré *et al*.^33^, where CsrA acts as a posttranscriptional regulator affecting RNA stability, but mediates much of its indirect effects on mRNA abundance though transcriptional effects controlled by CsrD. Bold arrows emphasize strong contributions to global gene expression in the models. Dashed lines indicate connections with limited experimental evidence. Dashed lines connecting CsrD and CsrA in Model 1 were proposed from the results in this study.

The Csr (Rsm) global regulatory system is conserved among *Gammaproteobacteria*^1,2^. It affects gene expression involved in major bacterial lifestyle decisions, and has been extensively studied for its roles in glycogen metabolism^3,4^, biofilm formation^5–7^, motility^8,9^, and virulence^1,10^. In *Escherichia coli*, the Csr system includes the RNA binding protein CsrA, two small regulatory RNAs (sRNAs), CsrB and CsrC (CsrB/C), which bind to and antagonize CsrA, and a protein that is specifically required for the turnover of CsrB/C, CsrD (Fig. 1a)^1^. Direct effects of CsrA on gene expression involve its binding to RNA sequences containing conserved GGA motifs in the 5′-untranslated or early coding region of mRNAs, leading to changes in RNA structure^11^, transcription elongation^12^, RNA stability^7,9,13^, and/or translation initiation^3,14,15^. CsrA can also regulate gene expression indirectly by controlling other regulators. For example, CsrA represses translation initiation of the transcription factor NhaR, which activates transcription of the *pgaABCD* operon^16,17^. Thus, CsrA indirectly represses transcription of *pgaABCD*, which is required for biofilm formation^7,16,17^. CsrA also directly regulates expression of FlhDC^8,9^, Hfq^18^, RelA^19^, DksA^19^, RpoE^20^, IraD^15^, PNPase^21^, and many other regulators that have not been investigated in detail^22^.

CsrA activity is controlled primarily by the steady state levels of CsrB/C, which contain many high affinity CsrA binding sites that facilitate sequestration of CsrA away from its lower affinity mRNA targets^1,23,24^. Transcription of CsrB/C is activated by the BarA-UvrY two-component regulatory system (TCS) in response to the accumulation of end products of metabolism, including acetate and formate^2,25^. Degradation of CsrB/C is initiated by the housekeeping endonuclease RNase E. CsrB/C turnover also requires the presence of CsrD and is stimulated by glucose via the interaction of unphosphorylated EIIA^Glc^ with CsrD (Fig. 1a)^26–28^. CsrD is a membrane-bound protein that contains degenerate GGDEF and EAL domains, which do not appear to synthesize, degrade, or respond to c-di-GMP^26^. Its EAL domain binds specifically to EIIA^Glc^ ^28^. Loss of CsrD strongly stabilizes CsrB/C but only modestly affects their steady state levels^26,29^, which is due to a negative feedback loop in which CsrA indirectly activates the transcription of these sRNAs via the BarA-UvrY TCS (Fig. 1a)^26,29–32^.

CsrD was initially described as a regulator of CsrB/C turnover and was found to regulate biofilm formation and *csrB* transcription in a CsrA- and CsrB/C- dependent manner^26^. These and other findings led to the development of a model for the workings of the Csr system, in which CsrD affects gene expression through changes in CsrB/C levels, which affect CsrA activity (Model 1, Fig. 1a). According to this model, CsrA acts as the most downstream regulator of gene expression in the Csr system. A recent transcriptomics study found that in addition to global effects of CsrA on RNA stability and steady state levels, CsrD exhibited opposing global effects on RNA levels without affecting stability^33^. Because the original model for the Csr system predicts that disruption of *csrA* and *csrD* should have similar effects on gene expression (Fig. 1a), an alternative model for the Csr system was formulated (Model 2, Fig. 1b). According to Model 2, CsrD is able to globally impact gene expression via transcriptional effects that are mediated downstream of CsrA, implying that CsrD can act as the terminal regulatory element of the Csr system. These two models make different predictions about the behavior of the Csr system and how it affects target gene expression on a global scale.

The goal of this study was to examine the relationships among the Csr system components CsrA, CsrB/C and CsrD in globally regulating gene expression. Epistasis analysis was used to determine the order of their genetic effects, which were assessed by using RNA-seq, an approach that we term Epi-seq. To determine whether the effects of CsrD on gene expression require CsrA and CsrB/C, we compared the impact of a *csrD* null mutation on the transcriptome in isogenic wild type (WT), *csrA* or *csrB/C* mutant strains. Likewise, CsrA effects on RNA abundance were assessed in strains with or without *csrD*. Our results demonstrate that under our batch culture conditions, which differed from the continuous culture conditions of the previous RNA-seq study^33^ the Csr circuitry functions as originally proposed (Fig. 1a), wherein CsrA is required for effects of CsrD on transcript levels. Nevertheless, CsrD may act in a limited capacity outside of CsrB/C, via unresolved mechanism(s), possibly through effects on decay of other RNAs besides CsrB/C.

## Results and Discussion

### Characterization of the wild type and mutant strains

**Figure 2:**
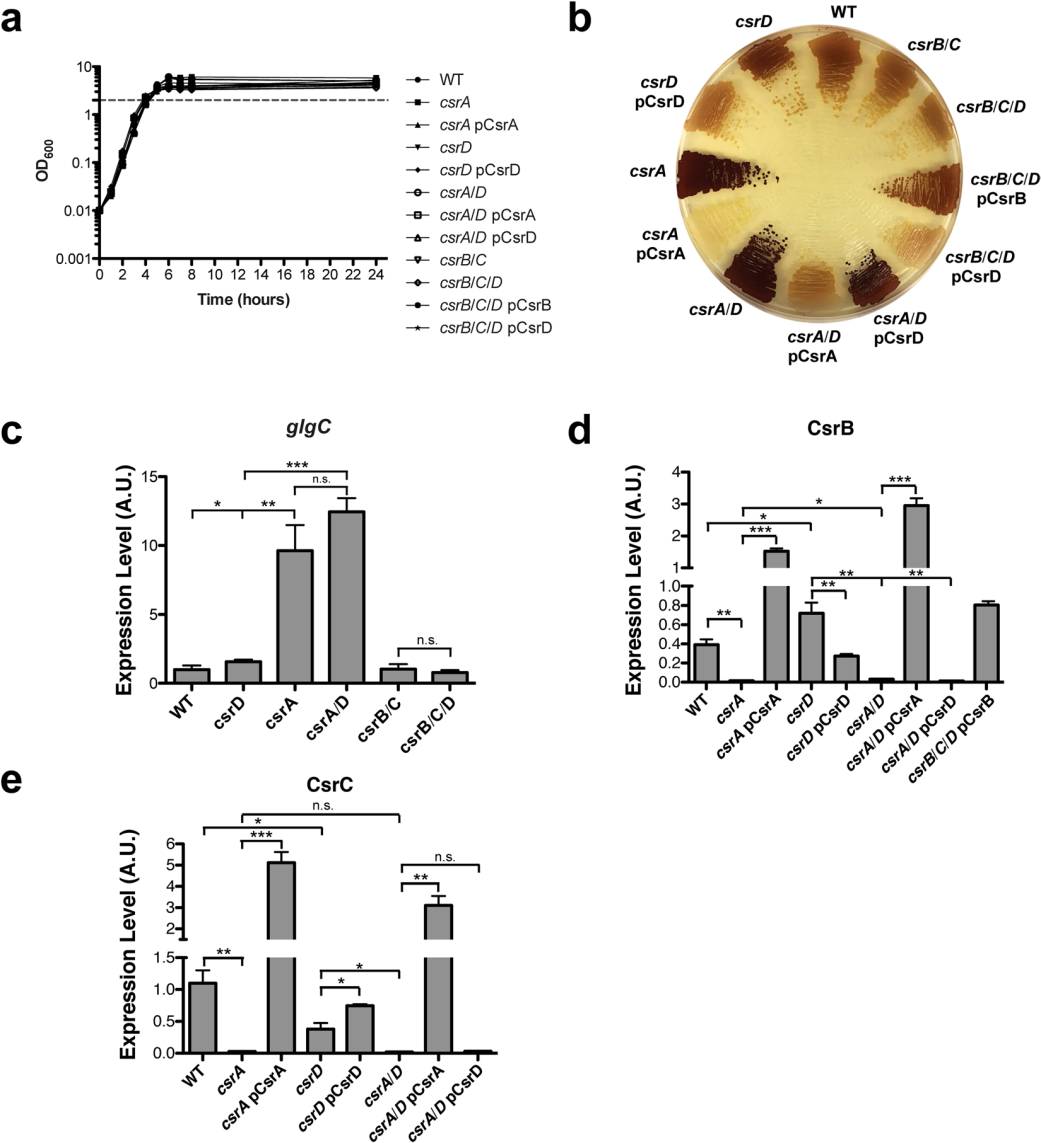
Phenotypes of bacterial strains used in this study are consistent with previous studies. (**a**) Growth curves of wild type (WT) and mutant strains carrying plasmids pBR322 (not labeled), pCsrA, pCsrD or pCsrB in Kornberg (KB) medium at 37 °C. (**b)** Glycogen production, determined by iodine staining. (**c**–**e)** qRT-PCR analysis of the transcript levels of *glgC*, *csrB*, and *csrC* at the transition to stationary phase of growth in KB medium. Error bars represent s.e. of the mean of three biological replicates. Asterisks indicate level of significance of a two-tailed student’s t-test (*p ≤ 0.05, **p ≤ 0.01, ***p ≤ 0.001).

To minimize the effect of growth rates on gene expression and allow comparative transcriptomic analysis between strains, we used the *csrA::gm* gene disruption mutant as the *csrA* mutant in this study^27^. This strain expresses a functionally impaired CsrA protein with greatly reduced affinity for RNA^9^. Unlike a *csrA* deletion strain which exhibits severe growth defects and rapidly accumulates suppressor mutations, this *csrA* mutant grows similarly to the WT strain in rich media^9,34,35^. This *csrA* allele is similar to the partial gene deletion (*csrA::51* allele) used previously^33^. The *csrB*, *csrC*, and *csrD* mutations were each complete gene deletions. As *csrA*/*D* double mutant strains showed dramatically enhanced cell aggregation and biofilm formation in liquid media (data not shown), all of the strains used in this study were constructed in a *pgaC* disruption mutant background^36^. The aggregative phenotype arises as a result of direct and indirect repression of *pgaABCD* expression by CsrA^7,16,37^. This operon encodes proteins required for biosynthesis and secretion of the polysaccharide adhesin poly-β-1,6-N-acetyl-D-glucosamine (PGA), and a *pgaC* mutant strain cannot synthesize PGA^36,38^. All strains carried an empty pBR322 plasmid or a pBR332-derivative encoding the appropriate gene for complementation analysis. Growth of all strains used in this study was essentially identical during the transition to stationary phase of growth in Kornberg (KB) medium at 37  °C, which was the condition used for the transcriptomics analyses (Fig. 2a)^39^.

To confirm that the strains used in this study behave consistently with previously reported effects of the Csr system, we analyzed glycogen levels and *glgC* expression. CsrA represses glycogen accumulation by reducing expression of the *glgCAP* operon, a phenotype that has been long associated with CsrA^4,13^. As expected, the *csrA* mutant accumulated far more glycogen and *glgC* mRNA relative to the WT strain, whereas deletion of *csrD* resulted in a slight increase in glycogen and a modest but significant increase in *glgC* mRNA levels relative to the WT strain (Fig. 2b,c). These effects on glycogen levels were both complemented by ectopic expression of the respective genes (Fig. 2b). Ectopic expression of *csrA* in the *csrA*/*D* mutant strain and *csrB* in the *csrB*/*C*/*D* mutant strain significantly reduced and stimulated glycogen levels, respectively (Fig. 2b). However, ectopic expression of *csrD* was unable to complement effects on glycogen levels in the *csrA*/*D* background (Fig. 2b). Finally, ectopic expression of *csrD* had little to no effect on glycogen levels in the *csrBCD* strain (Fig. 2b). These results are consistent with previously reported effects of the various Csr system components on glycogen levels^4,26^.

Due to the complexity of the feedback loops governing CsrB/C levels (Fig. 1a), we measured these RNAs in the strains that were used in this study. As described previously^29^, disruption of *csrA* in either the WT or *csrD* mutant strain significantly reduced CsrB and CsrC levels, and their expression was restored by ectopic expression of *csrA* (Fig. 2d,e). While CsrD is essential for normal turnover of CsrB/C^26^, deletion of *csrD* only moderately increased CsrB and decreased CsrC steady state levels (Fig. 2d,e). These phenotypes were complemented by ectopic expression of *csrD* (Fig. 2d,e). The opposing effect of CsrD on CsrC expression is most likely a result of an increase in CsrB levels in the *csrD* mutant. CsrB is the principal sRNA antagonist of CsrA, and CsrA indirectly activates CsrB and CsrC transcription through the BarA-UvrY TCS^1,3,24,26,29^. The compensatory effects of these RNAs on each other’s expression that result from this circuitry are well documented^24,33^. Thus, increased CsrB levels in the *csrD* mutant reduce CsrA activity, thereby decreasing CsrC transcription (Fig. 1a). Overall, these observations are consistent with established regulatory circuitry (Fig. 1a), wherein negative feedback loops affect both the synthesis and turnover of CsrB/C.

### CsrA retains its role as a global regulator in the absence of CsrD

**Figure 3:**
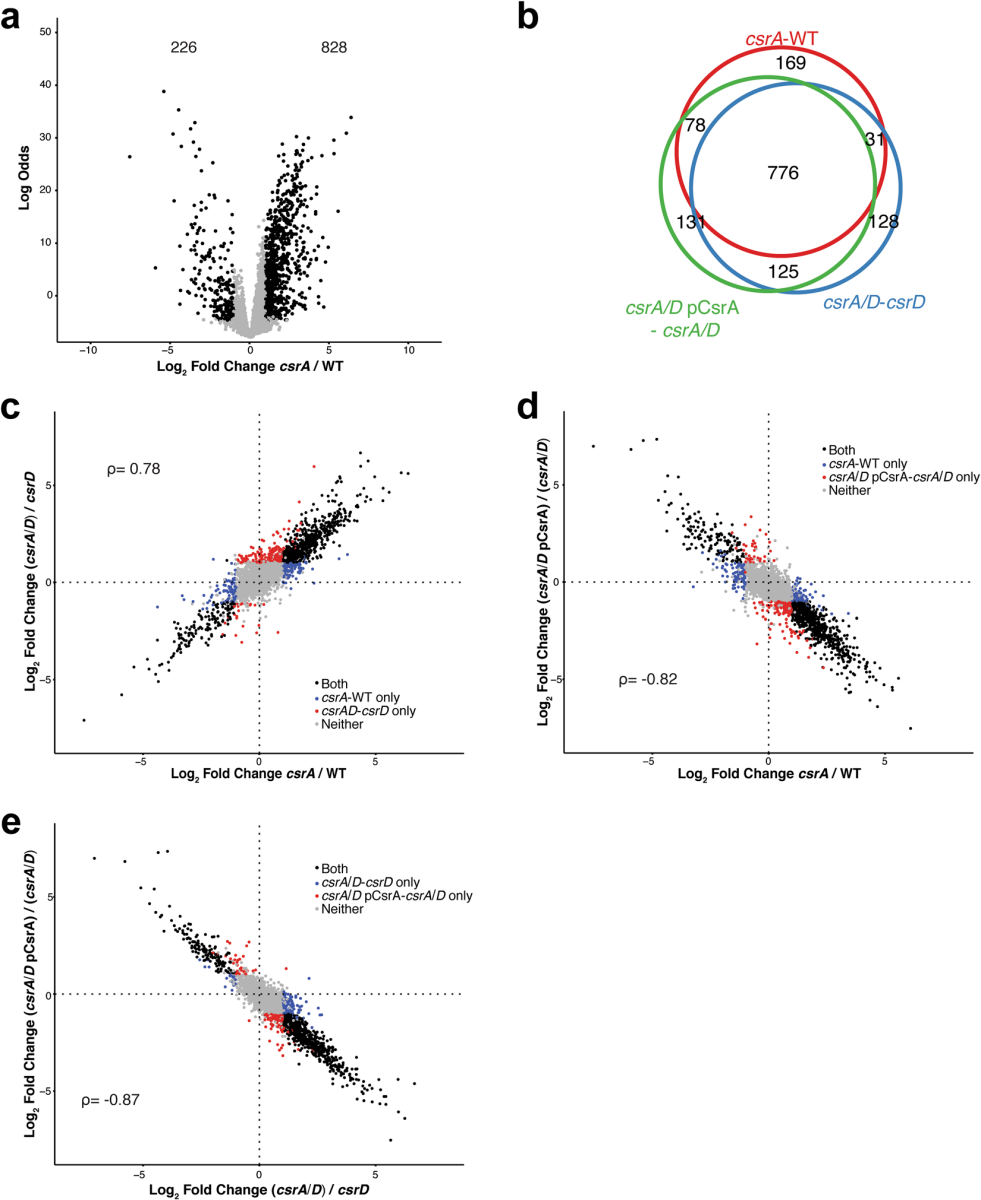
CsrA retains its global role in regulating mRNA levels in the absence of CsrD. (**a**) Volcano plot depicting the log_2_ transformed fold change of RNA levels between the *csrA* mutant and its isogenic WT strain versus log odds of significance. Genes with significant changes shown in black. The number of genes up- and down-regulated in this comparison are shown at the top of the plot. (**b**) Venn diagram depicting the overlap of genes differentially expressed among the following comparisons: *csrA* - WT, *csrA*/*D* - *csrD*, and *csrA*/*D* pCsrA - *csrAD*. (**c**–**e**) The log_2_ transformed fold change in RNA abundance caused by mutation or overexpression of *csrA* in the WT or *csrD* mutant backgrounds. Blue and red dots represent the genes that are only differentially expressed in one strain background. Black and grey dots represent the genes that are differentially expressed in both of the backgrounds or neither, respectively. The associated Spearman’s correlation coefficients (ρ) are shown.

Having established the strain set for the epistasis analysis, we next used RNA-seq to answer several core questions about the relationships between components of the Csr system. Differential expression analysis revealed 1,054 genes with significant changes in RNA abundance between the WT and *csrA* mutant strains (*csrA* - WT, Fig. 3a,b). CsrA repressed the expression of 828 genes and activated 226 genes (Fig. 3a). In addition, 807 out of these 1054 genes were differentially expressed between the *csrA*/*D* double mutant strain and the *csrD* mutant strain (*csrA*/*D* - *csrD*, Fig. 3b). This suggested that CsrA retains its global influence on 80% of its target genes in the absence of CsrD. We hypothesize that the remaining 20% of genes were not identified in this analysis due to the differences in CsrB/C levels resulting from the *csrD* mutation. These differences in CsrB/C levels would lead to changes in the amount of free CsrA, resulting in differences in the effect of CsrA on gene expression. We also compared the log_2_ transformed fold change in RNA abundance caused by the *csrA* mutation in the WT (*csrA* - WT) and *csrD* mutant strain (*csrA*/*D* - *csrD*) backgrounds. A relatively high Spearman’s correlation coefficient demonstrated that the absence of *csrD* had little impact on the overall magnitude of CsrA’s effect on gene expression (ρ = 0.78, Fig. 3c). Ectopic expression of *csrA* in the *csrA*/*D* double mutant resulted in vast changes in gene expression (*csrA*/*D* pCsrA - *csrA*/*D*) that were largely overlapping with those resulting from mutation of *csrA* in the WT strain (*csrA* - WT, Fig. 3b). The log_2_ transformed fold changes from ectopic expression of *csrA* in the *csrA*/*D* double mutant showed a strong negative Spearman’s correlation with those resulting from mutation of *csrA* in either a WT (*csrA* - WT, Fig. 3d) or *csrD* mutant strain background (*csrA*/*D* - *csrD*, Fig. 3e) (ρ = 0.82 and 0.87, respectively). Together these data indicate that CsrA does not require CsrD to exert global effects on transcript levels.

### CsrD effects on gene expression are lost in the *csrA* mutant

**Figure 4:**
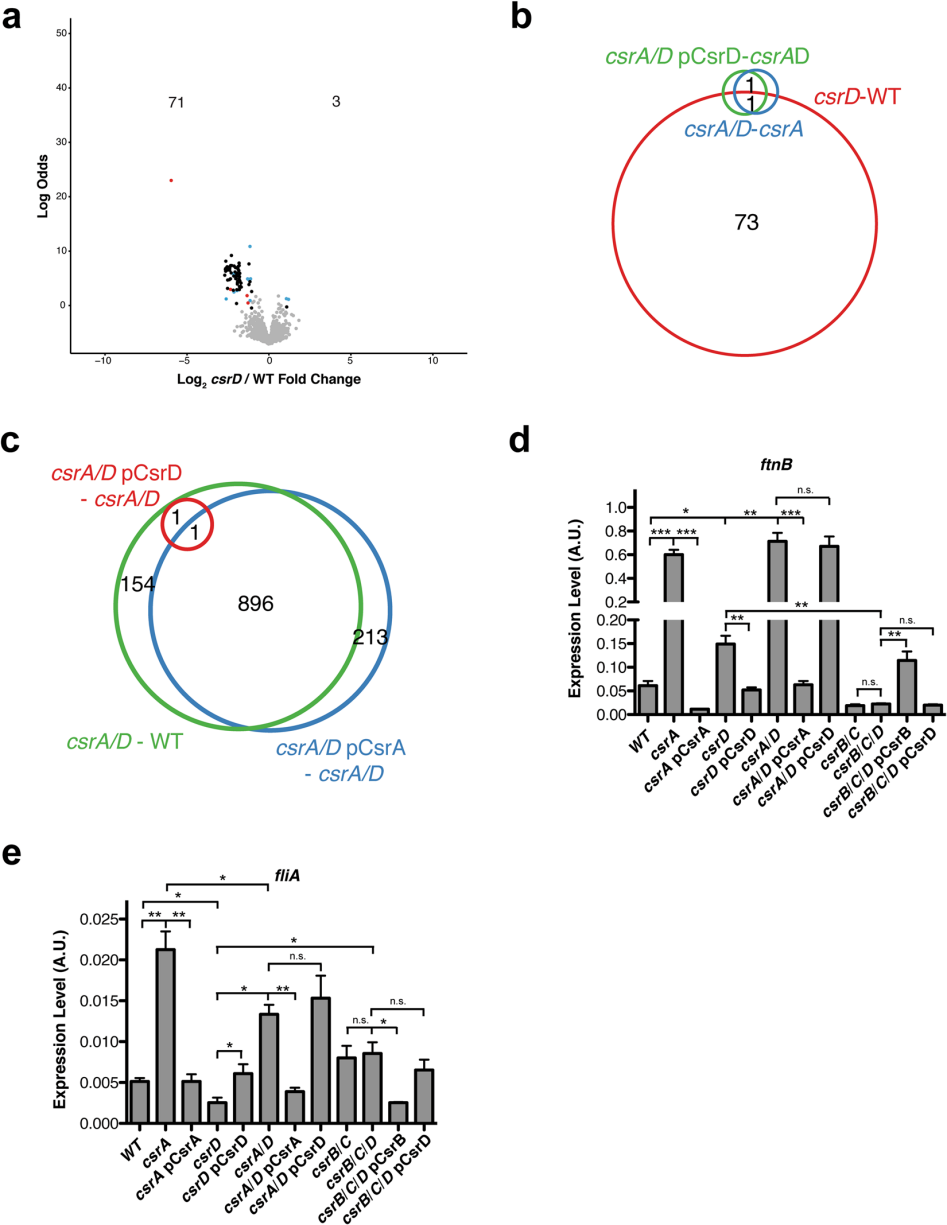
CsrD controls gene expression through CsrA-dependent pathways. (**a**) Volcano plot depicting the log_2_ transformed fold change of RNA levels between the *csrD* mutant and its isogenic WT strain versus log odds of significance. The number of genes up- and down-regulated in this comparison are shown at the top of the plot. Black dots represent genes that are only differentially expressed in *csrD* - WT but not *csrA* - WT comparison. Red dots represent genes that are regulated in the same direction in the *csrD* and *csrA* mutant strains relative to the WT strain. Cyan dots represent genes that are either upregulated or downregulated in both *csrD* and *csrA* mutant strains relative to the WT strain. Grey dots represent genes that are not differentially expressed in the *csrD* – WT comparison. (**b**) Venn diagram depicting the overlap of genes differentially expressed among the following comparisons: *csrD* - WT, *csrA*/*D* - *csrA*, and *csrA*/*D* pCsrD - *csrA*/*D*. (**c**) Venn diagram depicting the overlap of genes differentially expressed among the following comparisons: *csrA*/*D* - WT, *csrA*/*D* pCsrA - *csrA*/*D*, and *csrA*/*D* pCsrD - *csrA*/*D*. (**d–e)** qRT-PCR analysis of the transcript levels of *ftnB* and *fliA*. Error bars represent the s.e. of the mean of three biological replicates. Asterisks indicate level of significance of a two-tailed student’s t-test (*p ≤ 0.05, **p ≤ 0.01, ***p ≤ 0.001).

We identified 73 genes not including *csrD* that were differentially expressed between the WT and *csrD* mutant strains (*csrD* - WT, Fig. 4a), suggesting that CsrD has a limited effect on the transcriptome compared to CsrA under our experimental conditions (Figs 3a and 4a). In addition, the majority of these genes (54/73) are involved in motility and chemotaxis, suggesting that CsrD targets may exhibit limited functional roles. More critically, *csrB* was the only gene differentially expressed between the *csrA*/*D* double mutant and *csrA* mutant strains besides *csrD* (*csrA*/*D* - *csrA*, Fig. 4b), indicating that CsrD requires CsrA for essentially all of its effects on gene expression. In addition, ectopic expression of *csrD* was unable to complement the effects of the *csrA csrD* double mutant strain unlike ectopic expression of *csrA* in this strain (Fig. 4c). The clear inference from these findings is that *csrD* functions upstream of *csrA* to regulate gene expression.

The simplest interpretation of Model 1 (Fig. 1a) predicts that mutations in *csrA* and *csrD* would result in qualitatively similar changes in gene expression in the same direction. Indeed, CsrA regulated 12 genes affected by CsrD in the same direction (cyan in Fig. 4a). We used qRT-PCR to validate these results for one such gene, *ftnB* (Fig. 4d). Consistent with our RNA-seq data, both *csrA* and *csrD* mutations led to an increase in *ftnB* mRNA levels, and these effects were complemented by ectopic expression of the respective genes (Fig. 4d). Mutation of *csrA* in the *csrD* mutant background also resulted in increased *ftnB* levels, but there was no significant difference in *ftnB* levels between the *csrA* and *csrA*/*D* double mutant strains. In addition, ectopic expression of *csrA* but not *csrD* complemented the change in *ftnB* expression in the *csrA*/*D* double mutant strain (Fig. 4d). Taken together, these qRT-PCR results are consistent with the effects of CsrD being mediated through CsrA.

The remaining genes regulated by CsrD were either regulated by CsrA in the opposite direction (4 genes including *csrD*, red in Fig. 4a) or not regulated by CsrA (58 genes, black in Fig. 4a). Interestingly, nearly all of the latter genes were regulated by CsrA under a different condition (55/58)^22^. These results are more complex than can be explained with a simplistic interpretation of Model 1 (Fig. 1a). According to Model 2, opposing effects of CsrA and CsrD may be mediated through CsrD effects on transcription (Fig. 1b)^33^. Specifically, genes whose RNA stability was not affected by CsrA were proposed as possible candidates of CsrD-dependent transcriptional regulation^33^. To this end, we compared genes whose expression was regulated by CsrD in this study with an analysis of CsrA-dependent regulation of RNA stability^22^. Out of the 73 genes regulated by CsrD (*csrD*-WT, not including *csrD*), 49 of these were not regulated at the level of their RNA stability (18 were not analyzed, 2 were destabilized by CsrA and 4 stabilized by CsrA). Thus, it appears that many of the genes regulated by CsrD in our condition are not directly regulated at the level of RNA stability by CsrA, which would make them candidates for CsrD-dependent transcriptional regulation^33^. However, the epistasis analysis showed that *csrD* exhibited no effects on gene expression (other than *csrB* and *csrD* itself) in the absence of *csrA* (*csrA*/*D* - *csrA*, Fig. 4b). Nevertheless, we wanted to validate an example of possible CsrA-independent effects of CsrD on gene expression using qRT-PCR. CsrD activated the expression of *fliA*, which is consistent with RNA-seq data (Fig. 4e). Although it was not significant in the RNA-seq data, CsrA repressed *fliA* expression (Fig. 4e), which is consistent with previous evidence^22^. Both of these effects were complemented by the corresponding genes (Fig. 4e). Deletion of *csrD* in a *csrA* mutant background weakly affected *fliA* expression (20% decrease, Fig. 4e). In stark contrast, disruption of *csrA* in a *csrD* deletion mutant resulted in a large change in *fliA* expression (500% increase, Fig. 4e). In addition, ectopic expression of *csrA*, but not *csrD*, was able to complement the *csrA csrD* double mutant (Fig. 4e). The small effect still observed for the *csrD* deletion in the *csrA* disruption mutant likely occurs because this is not a null *csrA* allele. Finally, deletion of *csrD* did not affect *fliA* mRNA in the *csrB csrC* double deletion strain and ectopic expression of *csrB* but not *csrD* was able to complement the *csrB csrC csrD* triple mutant (Fig. 4e). Thus, even for genes such as *fliA*, for which *csrA* and *csrD* single mutations have opposing effects on gene expression, the effects of CsrD are mediated via the other Csr components. Although not as simplistic as either model implies (Fig. 1), these results suggest that small and large decreases in CsrA activity (*csrD* mutant and *csrA* mutant, respectively) may lead to different and even opposing effects on gene expression. As CsrA is a global regulator (Fig. 3a), perhaps it is not surprising that it can exhibit such complex effects on gene expression, reminiscent to what has been seen for DNA binding regulators, e.g. OmpR can activate or repress *ompF* at low or high osmolarity, respectively^40^.

### CsrB/C effects on gene expression are enhanced in the absence of CsrD

**Figure 5:**
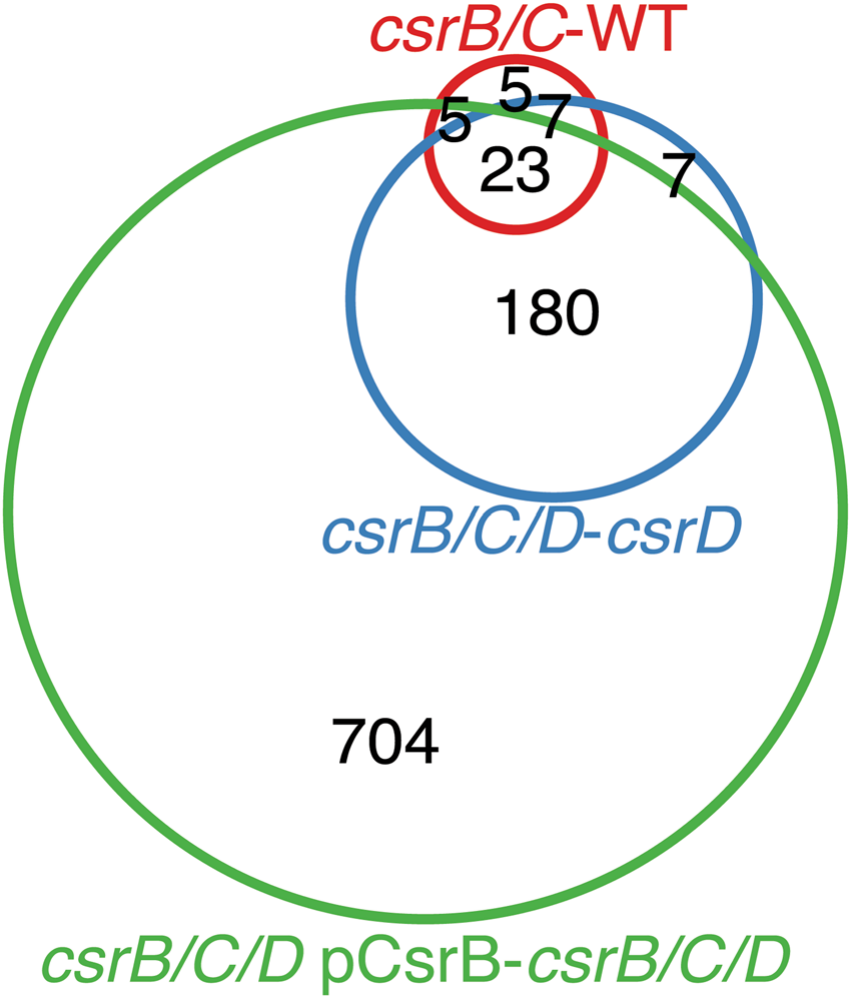
CsrB/C affect gene expression in the absence of CsrD. Venn diagram depicting the overlap of genes differentially expressed among the following comparisons: *csrB*/*C* - WT, *csrB*/*C*/*D* - *csrD*, *csrB*/*C*/*D* pCsrB - *csrB*/*C*/*D*.

CsrB/C affect gene expression indirectly by sequestering CsrA from interacting with its lower affinity mRNA targets^1,23,24^. If CsrA acts as the terminal factor in the Csr system, then CsrB/C should still impact gene expression in the absence of CsrD. On the other hand, if CsrD were able to act downstream of CsrA to regulate transcription, then its effects on transcription should also be predicted to persist in the absence of CsrB/C. Deletion of *csrB*/*C* resulted in changes in the expression of 40 genes in the WT background (*csrB*/*C* - WT) and 218 genes in the *csrD* mutant strain background (*csrB*/*C*/*D* - *csrD*) (Fig. 5). This finding confirmed that CsrB/C do not require CsrD to globally affect gene expression. More importantly, the greater effects of these sRNAs on expression in the *csrD* mutant compared to the WT background are as would be predicted if CsrD acts by triggering CsrB/C decay. Because of increased CsrB levels, CsrA activity in the *csrD* mutant strain should be lower relative to the WT strain (Fig. 2D). Consequently, CsrA activity is predicted to be more greatly affected by *csrB*/*C* deletion in the *csrD* mutant vs the WT background. The expression of 912 genes was significantly different when CsrB was ectopically expressed in the *csrB*/*C*/*D* mutant strain (*csrB*/*C*/*D* pCsrB - *csrB*/*C*/*D*, Fig. 5). This further demonstrates that CsrB mediates vast effects on gene expression in the absence of CsrD. Together with the observations that CsrA globally regulates gene expression in the absence of CsrD, while CsrD requires CsrA for its effects (Fig. 3), these findings reveal that neither CsrB/C nor CsrA requires CsrD to mediate global changes in gene expression.

### CsrD regulation of target genes is largely, but not entirely CsrB/C-dependent

**Figure 6:**
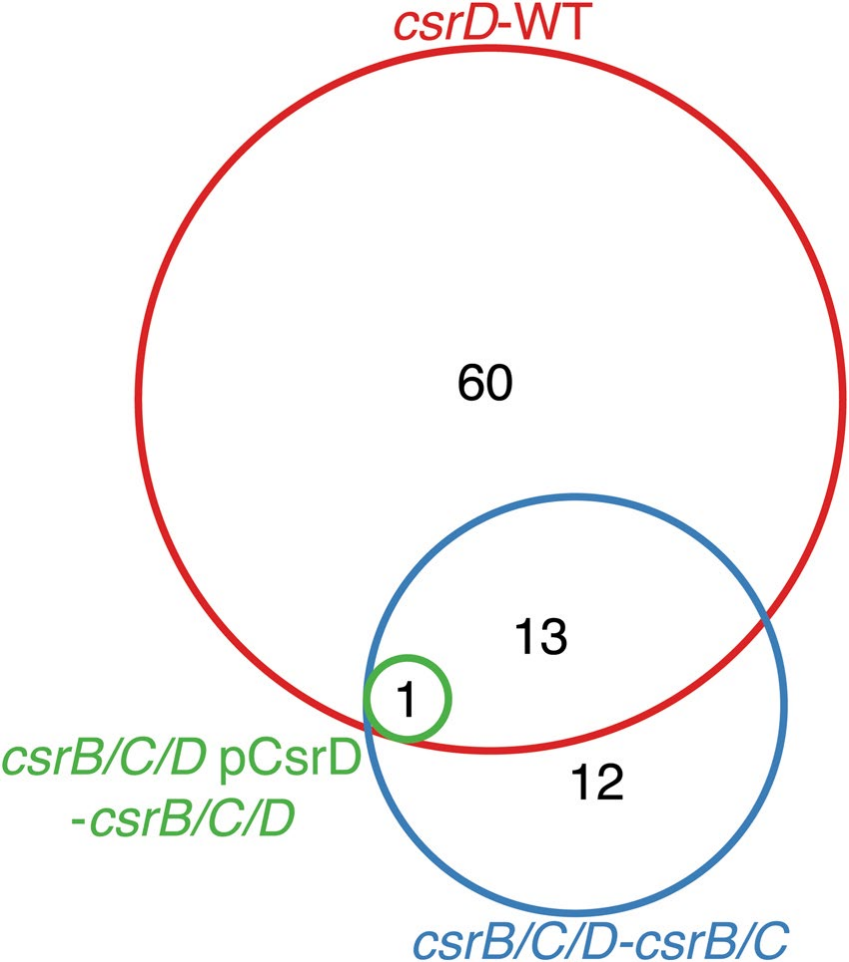
CsrD regulates gene expression in CsrB/C-dependent and independent pathways. Venn diagram depicting the overlap of genes differentially expressed among the following comparisons: *csrD* - WT, *csrB*/*C*/*D* - *csrB*/*C*, and *csrB*/*C*/*D* pCsrD - *csrB*/*C*/*D*.

To further determine whether CsrD always depends on CsrB/C to mediate changes in gene expression, we compared the impact of CsrD on the transcriptome in the presence and absence of CsrB/C. Differential expression analysis revealed 60 genes that respond to *csrD* deletion in the WT background (*csrD* - WT) but not in the *csrB*/*C* mutant background (*csrB*/*C*/*D* - *csrB*/*C*) (Fig. 6). Thus, 82% of the genes regulated by CsrD under our conditions are controlled via a CsrB/C-dependent pathway. Using qRT-PCR, we confirmed for one of these genes, *ftnB*, that CsrD no longer regulated its expression in the *csrB*/*C* mutant background (Fig. 4d). In the absence of CsrB/C, 25 genes not including *csrD* still responded to CsrD (*csrB*/*C*/*D* - *csrB*/*C*, Fig. 6), suggesting that alternative regulatory pathways may allow CsrD to mediate changes in expression. Intriguingly, we observed 12 genes that were regulated by CsrD only in the *csrB*/*C* mutant background (*csrB*/*C*/*D* – *csrB*/*C*, Fig. 6) but not in the WT background (*csrD* – WT, Fig. 6). This observation suggests that a CsrB/C-independent pathway might have an opposing effect on the expression of these genes with respect to the CsrB/C-dependent pathway. However, none of these 12 changes in expression were complemented by ectopic expression of *csrD* (*csrB*/*C*/*D* pCsrD – *csrB*/*C*/*D*, Fig. 6), whereas overexpression of *csrB* complemented most of the differences identified in a *csrB*/*C*/*D* triple mutant (*csrB*/*C*/*D* pCsrB - *csrB*/*C*/*D*, Fig. 5). Further investigation is required to determine the relevance of these differences. A plausible explanation for CsrB/C independent effects is that CsrD may regulate the stability of other sRNAs that regulate CsrA activity. For example, the sRNAs McaS and GadY have been shown to inhibit CsrA activity when overexpressed^41,42^. Recently other sRNAs have been found to directly bind CsrA *in vivo* in both *Samonella* and *E*. *coli*^22,43^. This hypothesis will require further testing. Nevertheless, a majority of CsrD effects are mediated through CsrB/C.

### Indirect regulatory role of CsrA

If CsrA does not mediate vast transcriptional effects on gene expression through CsrD as proposed (Fig. 1b), this raises the question as to how CsrA affects the transcriptome on such a global scale and in the absence of effects on stability^33^. In addition to global post-transcriptional regulation mediated through direct binding interactions, CsrA also indirectly regulates transcript levels by controlling the expression of transcriptional and post-transcriptional regulators^22^. Indeed, our data revealed that CsrA affects the expression of 68 transcriptional regulators, including TCS, σ factors, and other transcription factors (Supplementary Table S1). This supports other studies, which have suggested that CsrA interacts with many mRNAs encoding transcriptional regulators *in vivo*^19,22,43,44^. CsrA also affected the abundance of 8 sRNAs in addition to CsrB/C (Supplementary Table S2). sRNAs have been shown to contribute directly and indirectly to changes in transcript levels by altering mRNA turnover and the expression of transcriptional regulators^45,46^. Clearly, the integration of post-transcriptional regulation into transcriptional regulatory networks is a common theme used by bacteria to control their gene expression^45,46^. As result, post-transcriptional regulators including CsrA and sRNAs can mediate vast effects on gene expression indirectly, which allows them to control a broad range of genes and cellular functions. Thus, CsrA no doubt mediates changes in the levels of many transcripts indirectly through its effects on transcription factors and other regulators.

## Conclusions

Recent work has proposed that CsrA may exert much of its impact on the transcriptome via its effects on CsrD, which acts downstream of CsrA as a regulator of gene expression^33^. However, these studies rely heavily on correlational analysis of gene expression between single *csrA* and *csrD* mutant strains. On the other hand, existing models propose that CsrA is the terminal regulator in the Csr system with CsrD playing a role in gene expression solely through effects on CsrA activity^26^. However, the latter studies rely primarily on data from selected single gene analyses and may not capture the full effects of the Csr system on gene expression at a transcriptome-wide scale. Here we formally analyzed the relationship between different components of the Csr system on a whole genome level using epistasis analysis with RNA-seq (Epi-seq). We now provide evidence that CsrA and CsrB/C mediate vast changes in RNA abundance independently of CsrD. Moreover, CsrD affects gene expression largely through CsrB/C and seemingly entirely through CsrA. In addition, CsrD may affect expression of a limited number of genes independently of CsrB/C. The latter observation warrants further investigation, and hints that CsrD may affect the turnover of RNAs in addition to CsrB/C. Altogether, the use of Epi-seq to clarify the genetic relationships among the components of the Csr system indicates that CsrA acts as the terminal component of the Csr system to globally regulate gene expression. Because the present and previous^33^ studies used different experimental conditions, batch culture in Kornberg medium and *ΔpgaC* background vs. continuous culture in M9 glucose medium, respectively, it is conceivable that the Csr system functions differently under the two conditions. Thus, we acknowledge the formal possibility that CsrD might mediate CsrA-independent transcriptional effects under the latter growth conditions.

## Methods

### Media and growth conditions

Bacterial strains used in this study are listed in Supplementary Table S3. Strains were routinely grown in LB medium (1% tryptone, 1% NaCl, and 0.5% yeast extract) with antibiotics when appropriate: ampicillin (100 μg ml^−1^), kanamycin (50 μg ml^−1^), chloramphenicol (25 μg ml^−1^), and gentamicin (10 μg ml^−1^). For growth curve and RNA-seq analysis, overnight cultures grown in LB broth were inoculated into Kornberg medium (1.1% K_2_HPO_4_, 0.85% KH_2_PO_4_, 0.6% yeast extract and 0.5% glucose) at an OD_600_ of 0.01, and cultures were then grown at 37 °C with shaking at 250 rpm. Bacterial growth was monitored at OD_600_.

### Construction of strains and plasmids

*E*. *coli* K-12 MG1655 *pgaC880::cam* was used as the WT strain, where the *pgaC* gene was disrupted by a mini-Tn10 transposon with a *cam* resistance casette^36^. *E*. *coli* gene deletions and disruptions were transferred by P1*vir* transduction using *E*. *coli* donor strains from previous studies^26,27,36^ and the Keio library^47^, as shown in Supplementary Table S3. The FRT (short flippase recognition target)-flanked antibiotic resistance cassettes introduced into mutant strains were eliminated using an flippase expression plasmid pCP20 when necessary^48^.

Plasmids and DNA oligonucleotides used in this study are listed in Tables S4 and S5. Plasmids p2VR112 (referred as pCsrA in this study) and pBRY4 (referred as pCsrD in this study), express *csrA* and *csrD*, respectively, under the control of their native promoters on plasmid pBR322^26,27^. To construct plasmid pCsrB for expression of CsrB, the *csrB* gene with 494 base pairs (bp) upstream and 36 bp downstream was amplified from the genomic DNA and cloned into plasmid pBR322. Strains not transformed with pCsrA, pCsrD or pCsrB were transformed with pBR322 to maintain isogenic comparisons.

### Analysis of glycogen levels

Glycogen levels were analyzed by staining colonies with iodine vapor, as described previously^23^.

### RNA extraction and purification

During transition to stationary phase of growth (OD_600_ of 2.0), 1 ml of cell culture was collected and immediately mixed with 0.125 mL of stop solution (10% phenol/90% ethanol) to stabilize the RNA. Total RNA was isolated using hot phenol chloroform extraction followed by ethanol precipitation. Genomic DNA was removed by treating 20 μg of nucleic acid with 4U of Turbo DNase (Ambion), and RNA was purified from these reactions with the RNeasy kit (Qiagen). The integrity of the RNA was verified using denaturing gel electrophoresis and the RNA Bioanalyzer (Agilent).

### RNA-seq library preparation

For each strain, three independent biological replicates were collected. Ribosomal RNA was depleted from 5 μg of total RNA using the Ribo-Zero rRNA Removal Kit for Gram-Negative Bacteria (Illumina). The concentrations of the rRNA-depleted samples were determined with the Qubit RNA HS Assay Kit (ThermoFisher). RNA-seq libraries were then generated with the Stranded RNA-Seq Library Preparation Kit for Illumina (KAPA) and NEBNext Multiplex Oligos for Illumina adaptors (NEB) according to the manufacturer’s instructions for 100 ng of starting material and a mean insert size of 200–300 bases. Final libraries were purified with Pure Beads (KAPA). Sequencing library size and integrity were verified with DNA Bioanalyzer analysis (Agilent). Libraries were pooled and sequenced on 2 lanes of 50SE HiSeq. 2500 (Illumina) by the Genomic Services Laboratory at the HudsonAlpha Institute for Biotechnology.

### RNA-seq data analysis

Raw reads were demultiplexed and mapped to the *E*. *coli* rRNA sequences with Bowtie 2^49^. Unmapped rRNA depleted reads were then mapped to the *E*. *coli* genomic DNA sequence (NC_000913.3) with Bowtie 2^49^. Read counts per gene were calculated with htseq-count^50^. Read counts were filtered to remove genes with less than an average of 10 reads per sample across all samples. Differential expression was analyzed with limma voom^51^; fold changes >2 and a FDR (false discovery rate) <0.05 were considered significant. The full results are presented in Supplementary Table S6.

### Quantitative Reverse Transcriptase PCR (qRT-PCR)

qRT-PCR was conducted using iTaq Universal SYBR Green One-Step Kit (Bio-Rad) and an iQ5 iCycler real time PCR system (Bio-Rad) according to the manufacturer’s instructions. Reactions of 10 μl contained 200 ng of RNA or DNA standard, 300 nM of each primer, iScript reverse transcriptase, and 1× iTaq universal SYBR Green reaction mix. Reactions were incubated for 10 min of RT at 50 °C, 1 min of denaturation and RT inactivation at 95 °C, and then 45 cycles of 10 sec of denaturation at 95 °C and 20 sec of annealing, extension, and imaging at 60 °C. Melt curve analysis was used to verify the specificity of the amplicons with the parameters: 95 °C for 1 min, 55 °C for 1 min, and increasing the temperature 0.5 °C/10 sec until reaching 95 °C. RNA abundances were determined relative to a standard curve of PCR products and normalized to 16 s rRNA levels.

### Statistical analysis

All statistical tests used in this paper were two-sided, and statistical significance is indicated by asterisks (*p ≤ 0.05, **p ≤ 0.01, ***p ≤ 0.001).

### Data availability

All datasets generated by this study are included in the Supplementary Information and/or will be uploaded to the GEO repository upon acceptance of the manuscript.

## Electronic supplementary material

Supplemental Tables
